# Determinants of depressive symptoms in older outpatients with cardiometabolic diseases in a Japanese frailty clinic: Importance of bidirectional association between depression and frailty

**DOI:** 10.1371/journal.pone.0281465

**Published:** 2023-02-13

**Authors:** Fumino Yorikawa, Joji Ishikawa, Yoshiaki Tamura, Yuji Murao, Ayumi Toba, Kazumasa Harada, Atsushi Araki

**Affiliations:** 1 Tokyo Metropolitan Geriatric Hospital and Institute of Gerontology, Center for Comprehensive Care and Research for Prefrailty, Tokyo, Japan; 2 Department of Cardiology, Tokyo Metropolitan Geriatric Hospital, Tokyo, Japan; 3 Department of Diabetes, Metabolism and Endocrinology, Tokyo Metropolitan Geriatric Hospital, Tokyo, Japan; College of Medicine and Sagore Dutta Hospital, INDIA

## Abstract

**Introduction:**

Frailty and depression may play important roles in the management of older patients with cardiometabolic diseases. We explored the determinants of depressive symptoms and their association with frailty among patients with cardiometabolic diseases (hypertension, diabetes, and atrial fibrillation) in a cross-sectional study.

**Methods:**

A total of 633 outpatients aged 65 years or older with cardiometabolic disease and suspected symptoms of frailty participated in this study. Depressive symptoms, physical activity, and social network were assessed using the Geriatric Depression Scale (GDS)-15, International Physical Activity Questionnaire, and Lubben Social Network Scale-6 (LSNS-6), respectively. Frailty was evaluated using the Kihon Checklist (KCL) based on the Comprehensive Geriatric Assessment (CGA), the modified Cardiovascular Health Study (mCHS), and the Clinical Frailty Scale (CFS). Binomial logistic regression analysis was used to examine the determinants of depressive symptoms and their association with frailty.

**Results:**

Depressive symptoms with GDS-15 scores ≥ 5 were present in 43.6% of the patients. In logistic regression, after adjusting for covariates, the determinants of depressive symptoms in all patients were lack of social network, low years of education, and frailty. In contrast, in logistic regression with frailty as the dependent variable, depressive symptoms were independently associated KCL-defined frailty (OR = 6.28, 95% CI: 4.13–9.55) and mCHS-defined frailty (OR = 2.66, 95% CI: 1.70–4.17), but not with CFS. Similarly, significant associations between depression and frailty were observed in patients with hypertension, diabetes, or atrial fibrillation.

**Conclusions:**

Lack of social networks, low education, and frailty based on the KCL and mCHS were important determinants of depressive symptoms in all patients. The relatively strong associations between depressive symptoms and frailty based on CGA in patients with hypertension, diabetes, or atrial fibrillation suggest that the assessment of depressive symptoms is of great importance in clinical practice in those patients at high risk of frailty.

## Introduction

Older adults are more likely to have cardiometabolic diseases such as hypertension, diabetes, atrial fibrillation, and chronic heart failure. Patients with cardiometabolic disease are also more prone to depression. For example, patients with diabetes [1] are twice as likely to develop depression and survivors of acute myocardial infarction [2] are three times more likely to develop depression. Since depression in older adults with cardiovascular and metabolic diseases leads to increased incidence of cardiovascular events [3], increased mortality [4], and low adherence to self-care behaviors in patients with diabetes [5] prevention and early intervention for depression are important. In several studies using older general populations, depression has generally been associated with social isolation [6], physical functioning [7–9], socioeconomic factors [10], and cognitive impairment [11]. In some studies, the association between depression and age was attenuated in those aged over 55 years [12]. On the other hand, some of the factors associated with depression identified in the general elderly population have also been identified in the elderly with cardiovascular disease and metabolic disease, but the number of studies is small and cannot be generalized. For example, the main factors shown to be associated with depression in older diabetic patients are social isolation [13,14], cognitive impairment [15,16], reduced physical activity [17], and low income [18], but the number of studies is small and the association between social isolation and depression is inconsistent. In elderly hypertensive patients, low social support [19], low physical activity [17,20], and low income [19,20] have also been shown, but the number of studies is very small. In elderly patients with atrial fibrillation [21], studies are particularly scarce and only some associated factors have been examined. Furthermore, few studies have examined the determinants of depression in elderly patients with multiple cardiometabolic comorbidities.

In addition, depression is an important factor in the assessment of frailty, since depression in the elderly predicts frailty [22]. In the general population of older adults, frailty and depression are often comorbid [23,24] and a bidirectional relationship has been reported [23]: depressed people are more likely to be frail, and people with frailty are more likely to be depressed. This finding indicates that depression and frailty may be risk factors for each other. However, few studies have examined the association between depression and frailty in older patients with diabetes mellitus [25], heart failure [26], and atrial fibrillation [27].

Therefore, we compared the determinants of depression and the bidirectional association between depression and frailty among patients with cardiometabolic diseases, including hypertension, diabetes, and atrial fibrillation.

## Methods

### Participants

Between September 2015 and May 2021, we studied 633 older patients aged 65 years or attending the Department of Diabetes, Metabolism, and Endocrinology or the Department of Cardiology at our hospital who were suspected of having symptoms of frailty, such as decreased walking speed and fatigue, and visited the frailty clinic.

### Demographic and medical information

We assessed age, sex, years of education, number of medications taken, smoking status, drinking habits, antidepressant use, and underlying medical diagnoses based on information from medical interviews by physicians and medical records. The total number of diseases was the sum of hypertension, diabetes, dyslipidemia, stroke, heart failure, and atrial fibrillation.

### Geriatric assessment

Geriatric assessment, including questionnaire surveys by interviews and physical function tests of the patients, was conducted by a certified clinical psychologist to assess depressive symptoms, social support networks, physical activity, cognitive function, muscle strength, and frailty.

### Depressive symptoms

The Geriatric Depression Scale (GDS)-15 was used to assess depressive symptoms [28]. A score of ≥5 on the GDS-15 indicated the presence of depressive symptoms.

### Social networks

The shortened version of the Lubben Social Network Scale (LSNS) is a validated scale for screening social isolation among older adults. In this study, we also used the Japanese version of the LSNS-6 to assess the social isolation of the subjects [29]. The LSNS-6 consists of three questions that assess a patient’s relationships with relatives and friends. The questions were “How many relatives/friends do you see or hear from at least once a month?”; “How many relatives/friends do you feel close to such that you would call on them for help?”; and “How many relatives/friends do you feel at ease with that you can talk about private matters?”. For scoring, none = 0, one = 1, two = 2, three or four = 3, five–eight = 4, and nine or more = 5, with the total score on a scale of 0–30.

### Physical activity

To evaluate the amount of physical activity, we used the value of "total physical activity" in the short version of the International Physical Activity Questionnaire (IPAQ) [30]. Total physical activity (Mets × min/week) is a value that indicates the total amount of physical activity per week and is calculated by Mets × activity time per day × activity days per week. In accordance with the IPAQ guidelines, the values of total physical activity calculated using these equations were used as continuous variables in the analysis, and the median was adopted as the representative value for each group.

### Cognitive function

To evaluate cognitive function, we used the Japanese version of the Montreal Cognitive Assessment (MoCA-J), a cognitive function test to screen for mild cognitive impairment [31]. In our outpatient frailty clinic, we adopted the MoCA-J because patients showed a high prevalence of mild cognitive impairment.

### Muscle strength

To measure muscle strength, we assessed grip strength, which is believed to show a strong relationship with the overall muscle strength. In this study, one measurement was taken on each side and the maximum value was used.

### Frailty

The Kihon Checklist (KCL), modified Cardiovascular Health Study (mCHS) criteria, and Clinical Frailty Scale (CFS) were used to diagnose the presence of frailty. Eight points or more on the KCL [32], three points or more on the mCHS [33], and four points or more on the CFS [34] were considered to indicate the presence of frailty. The KCL consists of 25 questions assessing social participation, motor function, nutritional status, oral status, confinement, memory, and mental decline that can assess frailty based on the Comprehensive Geriatric Assessment (CGA). The mCHS was partially modified from the Cardiovascular Health Study (CHS) criteria [33]. In the mCHS, as in the CHS criteria, frailty was defined as muscle weakness (grip strength < 28 kg for males and < 18 kg for females), decreased walking speed (<1.0 m/s), fatigue, weight loss, and low physical activity. Walking speed was evaluated using the 4-m walk test. The questions on fatigue, weight loss, and decreased activity in the mCHS were taken from the KCL. Fatigue was defined as "I feel tired for no reason during the 2-week period, " and weight loss as "I have lost 2 kg or more of weight in the past 6 months”. Low physical activity was defined as answering “Yes” to either “Do you go out less often than once a week?” or “Have you gone out less often than last year?”.

### Ethical considerations

To participate in the study, an informed consent form was provided by the attending physician during the outpatient consultation, and the patient was considered a participant upon returning the consent form. This study was approved by the Ethics Committee of the Tokyo Metropolitan Geriatric Hospital (no. R15-20).

### Statistical analysis

Individuals with a GDS-15 score of ≥5 were defined as the group with depressive symptoms, and the frequency of depressive symptoms was investigated in this group. To compare demographic, medical, physical, psychological, and social variables between the depressed and non-depressed groups, we used two-sided t-tests or Mann–Whitney U tests for continuous variables and χ^2^ tests for dichotomous variables.

To examine the determinants of depressive symptoms, binomial logistic regression analysis with forced entry was performed for all patients and groups according to the presence of each cardiometabolic disease. The dependent variable was the presence or absence of depressive symptoms, and the independent variables were age, sex, and items that showed significant differences in univariate analysis: LSNS-6 score, years of education, number of medications, total physical activity, grip strength, hypertension, and frailty according to the KCL (Model 1), mCHS (Model 2), and CFS (Model 3). In the subgroup analysis, hypertension was removed from the independent variables.

In addition, binomial logistic regression analysis was performed to determine whether depressive symptoms could be a determinant of frailty in all patients and in each cardiometabolic disease, using the three types of frailty as dependent variables after adjusting for age, sex, LSNS-6, years of education, number of medications, total number of cardiometabolic diseases, total physical activity, and body mass index (BMI).

Variance inflation factors were less than 10 for the explanatory variables in these logistic regression analyses and did not show multicollinearity. Listwise deletion was used to exclude missing data from the analysis. IBM SPSS Statistics ver. 25 was used for the analysis, and statistical significance was set at p ≤ 0.05 was significant [35].

## Results

Participants’ characteristics are presented in Table 1. Mean participant age was 79.2 ± 6.4 years; 35.0% of the participants were men, and 3.3% were treated with antidepressants. The prevalence of diabetes, dyslipidemia, hypertension, heart failure, atrial fibrillation, and stroke was 50.7%, 60.5%, 73.6%, 8.0%, 10.5%, and 10.4%, respectively; MoCA-J, LSNS-6, and grip strength scores were 20.6 ± 4.9, 12.4 ± 5.8, and 21.0 ± 7.3 kg, respectively. The median total physical activity was 973 Mets × min/week. The prevalence of frailty in the three frailty indices was 35.0% for the KCL, 23.3% for the mCHS, and 33.8% for the CFS (Table 1).

**Table 1:** Characteristics of all participants and those with and without depressive symptoms.

		All participants	With depressive symptoms	Without depressive symptoms	test statistics	p-value
Variables		(n = 633)	(n = 276)	(n = 357)		
**Age**		79.2±6.4	79.5±6.6	78.9±6.2	-1.18	0.24
**Sex**	Men	222 (35.1%)	97 (35.1%)	125 (35.0%)	0.001	0.97
	Women	411 (64.9%)	179 (64.9%)	232 (65.0%)		
**Years of education**		**11.5±3.8**	**10.9±3.9**	**12.0±3.6**	**3.47**	**0.001**
**Smoking**		228 (36.0%)	105 (38.0%)	123 (34.4%)	1.04	0.60
**Alcohol habit**		124 (19.5%)	50 (18.1%)	74 (20.7%)	0.67	0.41
**BMI (kg/m^2^)**		23.2±3.7	23.0±4.0	23.3±3.4	1.26	0.21
**Number of medications/day**		**5.0 (3.0, 8.0)**	**6.0 (3.0, 8.0)**	**5.0 (2.5, 8.0)**	**2.69**	**0.007**
**Antidepressants**		**21 (3.3%)**	**15 (5.5%)**	**6 (1.7%)**	**6.84**	**0.009**
**Underlying disease**						
	Diabetes mellitus	321 (50.7%)	142 (51.4%)	179 (50.1%)	0.11	0.74
	Dyslipidemia	383 (60.5%)	163 (59.0%)	220 (61.6%)	0.43	0.51
	Hypertension	**466 (73.6%)**	**214 (77.5%)**	**252 (70.5%)**	**3.87**	**0.049**
	Heart failure	52 (8.0%)	21 (8.0%)	31 (9.0%)	0.24	0.63
	Atrial fibrillation	67 (10.5%)	26 (9.4%)	41 (11.4%)	0.70	0.40
	Stroke	66 (10.4%)	27 (9.7%)	39 (10.9%)	2.79	0.25
	Total number of cardiometabolic diseases	2.14±1.07	2.14±1.09	2.13±1.05	-0.08	0.93
**Cognitive function**	MoCA-J	20.6±4.9	20.4±4.8	20.7±5.0	0.81	0.42
**Muscle strength**	Grip strength (kg)	**21.0±7.3**	**20.0±7.0**	**22.2±9.9**	**3.03**	**0.002**
**Social network**	LSNS-6	**12.4±5.8**	**10.4±5.4**	**13.9±5.7**	**7.72**	**< 0.001**
**Total Physical activity**	IPAQ (Mets x min/week)	**973** **(354, 2011)**	**693** **(231, 1425)**	**1257** **(530, 2190)**	**-5.10**	**< 0.001**
**Frailty**	KCL (≥8)	**222 (35.0%)**	**163 (59.0%)**	**59 (16.5%)**	**123.65**	**< 0.001**
	mCHS (≥3)	**148 (23.3%)**	**98 (35.5%)**	**49 (13.7%)**	**40.90**	**< 0.001**
	CFS (≥4)	**214 (33.8%)**	**114 (41.3%)**	**100 (28.0%)**	**12.37**	**< 0.001**

The t-test was used for continuous variables and the χ^2^ test for binary variables; since number of medications/day and total physical activity (Mets × min/week) was not normally distributed, the Mann–Whitney U-test was used. In the t-test, χ^2^ test, and Mann-Whitney U-test, the test statistics are the t-value, χ^2^ value, and z-value, respectively. ±Indicates SD. (,) Indicates Quartile1, Quartile3.

The prevalence of depressive symptoms (GDS-15 ≥ 5) was 43.6% in all patients. The depressed group showed a higher number of medications, a higher prevalence of hypertension, and lower values for grip strength, gross motor activity, LSNS-6, and years of education. In patients with depressive symptoms, the prevalence of frailty was 59.0% for KCL, 35.5% for mCHS, and 41.3% for CFS, whereas the prevalence was 16.5% for KCL, 13.7% for mCHS, and 28.0% for CFS in those without depressive symptoms. The prevalence of frailty was significantly higher in those with depressive symptoms (Table 1).

To determine the determinants of depressive symptoms in all patients, binomial logistic regression analysis was performed with depressive symptoms as the dependent variable. Low LSNS-6 scores, short years of education, age, and KCL-defined frailty (OR = 6.35, 95% CI: 4.19–9.64) in Model 1, and LSNS-6 scores, years of education, and mCHS-defined frailty (OR = 2.49, 95% CI: 1.59–3.90) in Model 2 were independently associated with depressive symptoms (Table 2). In contrast, in Model 3, LSNS-6 scores, years of education, total physical activity, and grip strength, but not CFS-defined frailty, were independently related to depressive status.

**Table 2:** Binomial logistic regression analysis for the determinants of depressive symptoms in all patients with cardiometabolic disease.

	Model 1		Model 2		Model 3	
Independent Variables	OR (95% CI)	p-value	OR (95% CI)	p-value	OR (95% CI)	p-value
**LSNS-6 score**	**0.92(0.89–0.96)**	**<0.001**	**0.91(0.88–0.94)**	**<0.001**	**0.90(0.88–0.94)**	**<0.001**
**Years of education**	**0.93(0.89–0.98)**	**0.007**	**0.93(0.88–0.97)**	**0.002**	**0.92(0.88–0.97)**	**0.001**
**Number of medications**	1.03(0.97–1.09)	0.328	1.04(0.98–1.10)	0.179	1.05(0.99–1.10)	0.099
**Total physical activity**	0.99(0.98–1.01)	0.275	0.99(0.98–1.00)	0.104	**0.99(0.98–1.00)**	**0.035**
**Grip strength**	0.98(0.94–1.01)	0.213	0.97(0.93–1.00)	0.090	**0.97(0.94–1.00)**	**0.048**
**Age**	**0.95(0.92–0.98)**	**0.004**	0.98(0.95–1.01)	0.119	0.98(0.95–1.01)	0.251
**Sex**	1.21(0.73–2.01)	0.467	1.35(0.81–2.25)	0.253	1.27(0.79–2.05)	0.320
**Hypertension**	1.28(0.83–1.97)	0.264	1.13(0.74–1.71)	0.577	1.19(0.79–1.78)	0.401
**KCL (≥8)**	**6.35(4.19–9.64)**	**<0.001**	-	-	-	-
**mCHS (≥3)**	-	-	**2.49(1.59–3.90)**	**<0.001**	-	-
**CFS (≥4)**	-	-	-	-	1.28(0.87–1.88)	0.213

Logistic regression analysis was performed. Models 1, 2, and 3 included KCL, mCHS, and CFS, respectively, as independent variables as indicators of frailty. The value of total physical activity was calculated as the odds ratio per 100 Mets × min/week. Odds ratios (95% confidence intervals) are shown below. For Models 1, 2, and 3, the R^2^ were 0.318, 0.211, and 0.177, respectively.

The results of the logistic regression analysis showing the determinants of depressive symptoms for each disease are shown in Table 3. Depressive symptoms were present in 44.2% of the patients with diabetes, 45.9% of those with hypertension, and 38.8% of those with atrial fibrillation. Social isolation and frailty were selected as the determinants of depressive symptoms in patients with diabetes, hypertension, or atrial fibrillation. Years of education were selected as the determinants of disease-specific depressive symptoms in patients with hypertension and diabetes (Table 3). Among the diagnostic criteria for frailty, KCL-defined frailty was most strongly associated with depressive symptoms in any cardiometabolic disease.

**Table 3:** Binominal logistic regression analysis for the determinants of depressive symptoms in patients with hypertension, diabetes mellitus, or atrial fibrillation.

		Model 1			Model 2			Model 3		
**Independent Variables**		**HT** ^**†**^	**DM** ^**‡**^	**AF** ^**§**^	**HT** ^**†**^	**DM** ^**‡**^	**AF** ^**§**^	**HT** ^**†**^	**DM** ^**‡**^	**AF** ^**§**^
		**(n = 466)**	**(n = 321)**	**(n = 67)**	**(n = 466)**	**(n = 321)**	**(n = 67)**	**(n = 466)**	**(n = 321)**	**(n = 67)**
**LSNS-6**		**0.92** ***	**0.93** **	0.89	**0.90** ***	**0.92** ***	0.86*	**0.90** ***	**0.92** ***	**0.85** **
		**(0.88–0.96)**	**(0.89–0.98)**	(0.79–1.01)	**(0.86–0.93)**	**(0.88–0.96)**	**(0.76–0.97)**	**(0.87–0.93)**	**(0.88–0.96)**	**(0.76–0.96)**
**Years of education**		**0.95** *	0.95	0.97	**0.93** *	**0.92** *	0.98	**0.93** **	**0.92** *	0.99
		**(0.89–1.00)**	(0.88–1.01)	(0.82–1.14)	**(0.88–0.99)**	**(0.86–0.99)**	(0.83–1.16)	**(0.88–0.98)**	**(0.86–0.99)**	(0.84–1.16)
**Number of medications**		1.04	1.02	1.07	1.04*	1.04	1.12	1.05	1.04	1.13
		(0.97–1.11)	(0.94–1.11)	(0.87–1.32)	(0.97–1.11)	(0.97–1.13)	(0.92–1.36)	(0.98–1.11)	(0.97–1.12)	(0.92–1.39)
**Total Physical Activity**		0.99	0.99	0.99	0.99	0.99	0.99	**0.98** *	**0.98** *	0.98
		(0.98–1.00)	(0.97–1.00)	(0.92–1.06)	(0.97–1.00)	(0.97–1.00)	(0.93–1.06)	**(0.97–1.00)**	**(0.97–1.00)**	(0.92–1.05)
**Grip strength**		0.99	0.97	1.01	0.98	**0.95** *	1.10	0.98	0.96	1.02
		(0.95–1.03)	(0.92–1.02)	(0.90–1.14)	(0.94–1.03)	**(0.90–1.00)**	(0.94–1.26)	(0.94–1.02)	(0.92–1.01)	(0.92–1.14)
**Age**		**0.95** **	**0.94** *	1.01	0.97	0.97	1.04	0.97	0.97	1.01
		**(0.91–0.98)**	**(0.89–0.99)**	(0.91–1.13)	(0.94–1.01)	(0.92–1.01)	(0.93–1.16)	(0.94–1.01)	(0.93–1.01)	(0.92–1.12)
**Sex**		1.22	0.97	0.92	1.30	1.23	0.41	1.34	1.07	0.71
		(0.68–2.19)	(0.47–1.99)	(0.20–4.27)	(0.71–2.37)	(0.62–2.46)	(0.07–2.35)	(0.77–2.33)	(0.55–2.08)	(0.17–3.08)
**KCL (≥8)**		**5.75** ***	**7.00** ***	**5.14** *	-	-	-	-	-	-
		**(3.52–9.40)**	**(3.82–12.82)**	**(1.35–19.48)**						
**mCHS (≥3)**		-	-	-	**2.43** **	1.75	**5.43** *	-	-	-
					**(1.46–4.05)**	(0.94–3.26)	**(1.23–24.05)**			
**CFS (≥4)**		-	-	-	-	-	-	**1.58** *	1.35	1.49
								**(1.01–2.48)**	(0.79–2.30)	(0.43–5.18)

Logistic regression analysis was performed. Models 1, 2, and 3 included KCL, mCHS, and CFS, respectively, as independent variables as indicators of frailty. R^2^ in Model 1 were 0.307 in hypertensive patients, 0.328 in diabetic patients, and 0.356 in atrial fibrillation patients. Similarly, Model 2 were 0.213, 0.197, 0.322. Model 3 were 0.192, 0.179, 0.266. The total physical activity values were calculated as odds ratios per 100 Mets × minutes/week. Odds ratios (95% confidence intervals) are shown below. Sex indicates male versus female

†Hypertension

‡Diabetes

§Atrial fibrillation

*p ≤ 0.05

**p ≤ 0.01

***p ≤ 0.001.

We also examined whether depressive symptoms were independently linked to these three types of frailty (Table 4). The results showed that depressive symptoms were independently associated with frailty according to the KCL and mCHS in all patients and in those with each cardiometabolic disease. After adjusting for age, sex, LSNS-6 score, years of education, number of medications, total number of cardiometabolic diseases, physical activity, and BMI, depressive symptoms were associated with a higher prevalence of KCL-defined frailty in all patients (OR = 6.28, 95% CI: 4.13–9.55) as well as patients with hypertension (OR = 5.58, 95% CI: 3.39–9.19), diabetes (OR = 7.39, 95% CI: 3.99–13.67), and atrial fibrillation (OR = 4.79, 95% CI: 1.17–19.55). In a similar analysis, odds ratios of mCHS-defined frailty associated with depressive symptoms were 2.66 (95% CI: 1.70–4.17) in all patients, 2.38 (95% CI: 1.42–3.99) in those with hypertension, 1.92 (95% CI: 1.01–3.67) in those with diabetes, and 3.87 (95% CI: 1.04–14.47) in those with atrial fibrillation. Patients with heart failure could not be evaluated because of the small sample size. CFS frailty was not associated with depressive symptoms in any of the patients.

**Table 4:** Binominal logistic regression analysis for the association of depressive symptoms with three types of frailty in all patients and those with hypertension, diabetes mellitus, or atrial fibrillation.

	Total	HT^†^	DM^‡^	AF^§^
dependent variables	Odds ratio	Odds ratio	Odds ratio	Odds ratio
	(95% CI)	(95% CI)	(95% CI)	(95% CI)
**KCL (≥8)**	**6.28** ^***^	**5.58** ^***^	**7.39** ^***^	**4.79** *
	**(4.13–9.55)**	**(3.39–9.19)**	**(3.99–13.67)**	**(1.17–19.55)**
**mCHS (≥3)**	**2.66** ^***^	**2.38** **	**1.92** *	**3.87** *
	**(1.70–4.17)**	**(1.42–3.99)**	**(1.01–3.67)**	**(1.04–14.47)**
**CFS (≥4)**	1.3	1.57	1.32	1.2
	(0.87–1.92)	(0.99–2.48)	(0.76–2.29)	(0.31–4.67)

Logistic regression analysis was performed. Confounding factors were adjusted for age, sex, LSNS-6 scores, years of education, number of medications, total number of cardiometabolic diseases, total physical activity, and BMI. The total physical activity was calculated as the odds ratio per 100 Mets × minutes/week. Odds ratios (95% confidence intervals) are shown below. The R^2^ in the logistic regression analysis with KCL as the dependent variable were 0.392 in total, 0.423 in hypertension, 0.381 in diabetes, and 0.516 in atrial fibrillation. Similarly, in the analysis with mCHS as the dependent variable, 0.264, 0.267, 0.237, 0.328. In the analysis with CFS as the dependent variable, 0.236, 0.258, 0.220, 0.440.

†Hypertension

‡Diabetes

§Atrial fibrillation

*p ≤ 0.05

**p ≤ 0.01

***p ≤ 0.001.

## Discussion

In our study, lack of social networks, low education, and frailty were selected as determinants of depressive symptoms in older patients with cardiometabolic disease.

In particular, social isolation was an important determinant of depressive symptoms in all patients, including those with hypertension, diabetes, and atrial fibrillation, in this study, which is consistent with another study of the general population, diabetes patients and hypertension patients showing an association between low social support and depression [6,14,19]. Because the LSNS-6 in this study is an indicator that includes social support, such as emotional and instrumental support, the relative importance of social network size and network quality (social support) for the prevention of depressive symptoms remains unknown. However, since patients with cardiometabolic disease are likely to experience psychological distress and anxiety related to complications and adverse drug reactions, emotional support could be helpful in mitigating depressive symptoms [14,36]. Social support has been shown to result in better disease management through the alleviation of depressive symptoms. For example, Studies in older adults have shown that social support positively influences hypertension control by relieving depression and improving medication adherence [37]. In adults with diabetes, social support has also been shown to decrease depressive symptoms and promote self-care [38]. The prevention of depression and promotion of healthy behaviors through good social support may be synergistic.

In all patients and in patients with hypertension and diabetes, years of education were associated with depression, consistent with other studies [39] showing an association between education and depression in the older general population and in older patients with diabetes and hypertension [18,40]. Currently, few studies have examined the association between low education and depression in older adults with cardiovascular and metabolic diseases, but poor economic status in addition to fewer years of education may lead to poor adherence to self-care activities for illness [41] and poor adaptation to aging [42]. Our results suggest that older hypertensive and diabetic patients with lower levels of education are more likely to have depressive symptoms, probably because of their lower ability to cope with illness and poor adaptation to aging.

Our study showed that frailty was an important determinant of depressive symptoms in older patients with cardiometabolic diseases, which is consistent with previous studies using the general elderly population [23,43], diabetic patients [25] and patients with heart failure [26]. In the present study, frailty according to the KCL and CHS criteria, but not the CFS, was associated with depressive status. Individuals with KCL-defined frailty had a 6–7-fold higher odds ratio for depressive symptoms, which was higher than that of frail patients according to the CHS criteria. These results suggest that the CGA-based assessment of frailty is a better predictor of the presence of depression. The association between frailty assessment and depression by CGA is consistent with the general population of older adults [43]. Low physical activity, poor cognition, and lack of social activity due to multidimensional frailty promote depressive mood. The lack of association between depressive symptoms and CFS is inconsistent with a study of community-dwelling older adults aged 60 years and older [44]. These discrepancies may be explained by differences in target age, disease and drug treatment, and sociodemographic information.

On the contrary, depressive symptoms have also been found to be a determinant of frailty in older adults with cardiometabolic diseases. These results support the findings of longitudinal studies in the elderly general population [22] and cross-sectional studies in cardiometabolic diseases [25–27], indicating that depressive symptoms may be a risk factor for frailty in the elderly with cardiometabolic diseases. Disease-specific analyses yielded similar results, revealing that depressive symptoms such as hypertension, diabetes, and atrial fibrillation may increase the risk of frailty. In our study, the causal relationship between depression and frailty is unknown, but the association may be explained by mechanisms such as inflammation [45,46], hypothalamic-pituitary-adrenal (HPA) axis dysfunction [47,48], and cerebral small vessel disease [49,50], which are commonly found in the background of depression and frailty. High levels of pro-inflammatory cytokines, such as IL-6, CRP, cortisol [45–48], and cerebral white matter lesions [49,50] in patients with depression and frailty may adversely affect each other, leading to an increased risk of cerebrovascular disease, decreased activities of daily living, and mortality.

The strength of this study is that depressive symptoms and the three types of frailty were assessed in a relatively large sample of patients with three cardiometabolic diseases. The relatively strong associations between depressive symptoms and frailty based on CGA in any patient with hypertension, diabetes, or atrial fibrillation suggest that screening for depression is of great importance in clinical practice focusing on frailty in older patients.

This study also had several limitations. First, because this was a cross-sectional study, the causal relationship between depressive symptoms and frailty was unclear. Second, the results of the present single-center study should be confirmed by multicenter studies or other population-based surveys. Third, our study may have a selection bias because we recruited patients with suspected frailty, such as fatigue, which would have affected our results for the prevalence of depressive symptoms and frailty. Lastly, other confounding factors that were not analyzed in the present study, such as the intake of nutrients, inflammatory cytokines, and economic conditions, may have affected the association between depressive symptoms and frailty.

In conclusion, a lack of social networks, low education, and frailty were important determinants of depressive symptoms in older adults with cardiometabolic diseases. The relatively strong associations between depressive symptoms and frailty based on CGA in patients with hypertension, diabetes, or atrial fibrillation suggest that the assessment of depressive symptoms is of great importance in clinical practice in those patients at high risk of frailty. Future longitudinal studies are necessary to clarify whether interventions to reduce depressive symptoms, such as cognitive behavioral therapy and antidepressants, could prevent incident frailty in older patients with cardiometabolic diseases.

## Supporting information

S1 File Include detailed results of logistic regression analysis. (DOCX)
