# Supplementary material for: Determinants of depressive symptoms in older outpatients with cardiometabolic diseases in a Japanese frailty clinic: Importance of bidirectional association between depression and frailty
Source: PLoS One. 2023 Feb 13;18(2):e0281465. doi: 10.1371/journal.pone.0281465 (PMC9925076; doi:10.1371/journal.pone.0281465)
Supplement: S1 File — (DOCX) [file pone.0281465.s001.docx]

**Detailed results of the regression analysis are shown below.**

**Results of detailed binomial logistic regression analysis in Table 2.**

**Below are the results of Model 1 with depressive symptoms as the dependent variable.**

| **Hosmer and Lemeshow Tests** | | |
| --- | --- | --- |
| χ square | degree of freedom | p-value |
| 5.327 | 8 | 0.722 |

| **Variables in equations** | | | | | | | | |
| --- | --- | --- | --- | --- | --- | --- | --- | --- |
|  | B | SE | Wald | degree of freedom | p-value | Exp(B) | 95% confidence interval | |
|  |  |  |  |  |  |  | minimum | maximum |
| **LSNS-6 score** | -0.080 | 0.018 | 20.338 | 1 | 0.000 | 0.923 | 0.892 | 0.956 |
| **Years of education** | -0.068 | 0.026 | 7.204 | 1 | 0.007 | 0.934 | 0.888 | 0.982 |
| **Number of medications** | 0.028 | 0.029 | 0.957 | 1 | 0.328 | 1.029 | 0.972 | 1.088 |
| **Total physical activity** | -0.007 | 0.006 | 1.191 | 1 | 0.275 | 0.993 | 0.982 | 1.005 |
| **Grip strength** | -0.022 | 0.018 | 1.552 | 1 | 0.213 | 0.978 | 0.944 | 1.013 |
| **Age** | -0.049 | 0.017 | 8.273 | 1 | 0.004 | 0.952 | 0.921 | 0.985 |
| **Sex** | 0.189 | 0.260 | 0.528 | 1 | 0.467 | 1.208 | 0.725 | 2.013 |
| **Hypertension** | 0.246 | 0.220 | 1.249 | 1 | 0.264 | 1.279 | 0.831 | 1.970 |
| **KCL (≥8)** | 1.849 | 0.213 | 75.616 | 1 | 0.000 | 6.354 | 4.188 | 9.639 |

Sex indicates a man against a woman.

**Results of detailed binomial logistic regression analysis in Table 2.**

**Below are the results of Model 2 with depressive symptoms as the dependent variable.**

| **Hosmer and Lemeshow Tests** | | |
| --- | --- | --- |
| χ square | degree of freedom | p-value |
| 2.089 | 8 | 0.978 |

| **Variables in equations** | | | | | | | | |
| --- | --- | --- | --- | --- | --- | --- | --- | --- |
|  | B | SE | Wald | degree of freedom | p-value | Exp(B) | 95% confidence interval | |
|  |  |  |  |  |  |  | minimum | maximum |
| **LSNS-6 score** | -0.100 | 0.017 | 34.543 | 1 | 0.000 | 0.905 | 0.876 | 0.936 |
| **Years of education** | -0.075 | 0.025 | 9.261 | 1 | 0.002 | 0.928 | 0.884 | 0.974 |
| **Number of medications** | 0.037 | 0.028 | 1.806 | 1 | 0.179 | 1.038 | 0.983 | 1.096 |
| **Total physical activity** | -0.010 | 0.006 | 2.645 | 1 | 0.104 | 0.990 | 0.979 | 1.002 |
| **Grip strength** | -0.033 | 0.019 | 2.872 | 1 | 0.090 | 0.968 | 0.932 | 1.005 |
| **Age** | -0.025 | 0.016 | 2.431 | 1 | 0.119 | 0.975 | 0.945 | 1.006 |
| **Sex** | 0.298 | 0.261 | 1.304 | 1 | 0.253 | 1.348 | 0.808 | 2.249 |
| **Hypertension** | 0.118 | 0.212 | 0.311 | 1 | 0.577 | 1.125 | 0.743 | 1.705 |
| **mCHS (≥3)** | 0.913 | 0.228 | 16.038 | 1 | 0.000 | 2.493 | 1.594 | 3.898 |

Sex indicates a man against a woman.

**Results of detailed binomial logistic regression analysis in Table 2.**

**Below are the results of Model 3 with depressive symptoms as the dependent variable.**

| **Hosmer and Lemeshow Tests** | | |
| --- | --- | --- |
| χ square | degree of freedom | p-value |
| 6.098 | 8 | 0.636 |

| **Variables in equations** | | | | | | | | |
| --- | --- | --- | --- | --- | --- | --- | --- | --- |
|  | B | SE | Wald | degree of freedom | p-value | Exp(B) | 95% confidence interval | |
|  |  |  |  |  |  |  | minimum | maximum |
| **LSNS-6 score** | -0.100 | 0.017 | 36.535 | 1 | 0.000 | 0.905 | 0.876 | 0.935 |
| **Years of education** | -0.079 | 0.024 | 10.962 | 1 | 0.001 | 0.924 | 0.882 | 0.968 |
| **Number of medications** | 0.044 | 0.027 | 2.726 | 1 | 0.099 | 1.045 | 0.992 | 1.102 |
| **Total physical activity** | -0.013 | 0.006 | 4.465 | 1 | 0.035 | 0.987 | 0.976 | 0.999 |
| **Grip strength** | -0.033 | 0.017 | 3.894 | 1 | 0.048 | 0.967 | 0.936 | 1.000 |
| **Age** | -0.018 | 0.016 | 1.316 | 1 | 0.251 | 0.982 | 0.953 | 1.013 |
| **Sex** | 0.242 | 0.243 | 0.991 | 1 | 0.320 | 1.273 | 0.791 | 2.049 |
| **Hypertension** | 0.173 | 0.206 | 0.705 | 1 | 0.401 | 1.188 | 0.794 | 1.778 |
| **CFS (≥4)** | 0.244 | 0.196 | 1.550 | 1 | 0.213 | 1.277 | 0.869 | 1.876 |

Sex indicates a man against a woman.

**Results of detailed binomial logistic regression analysis in Table 3**

**Models 1, 2, and 3 with depressive symptoms as the dependent variable in hypertensive patients are as follows.**

**[Below are the results of the analysis for Model 1]**

| **Hosmer and Lemeshow Tests** | | |
| --- | --- | --- |
| χ square | degree of freedom | p-value |
| 2.794 | 8 | 0.947 |

| **Variables in equations** | | | | | | | | |
| --- | --- | --- | --- | --- | --- | --- | --- | --- |
|  | B | SE | Wald | degree of freedom | p-value | Exp(B) | 95% confidence interval | |
|  |  |  |  |  |  |  | minimum | maximum |
| **LSNS-6 score** | -0.084 | 0.020 | 16.995 | 1 | 0.000 | 0.919 | 0.883 | 0.957 |
| **Years of education** | -0.054 | 0.030 | 3.195 | 1 | 0.074 | 0.948 | 0.894 | 1.005 |
| **Number of medication** | 0.039 | 0.033 | 1.355 | 1 | 0.244 | 1.039 | 0.974 | 1.110 |
| **Total physical activity** | -0.010 | 0.007 | 2.142 | 1 | 0.143 | 0.990 | 0.976 | 1.004 |
| **Grip strength** | -0.011 | 0.021 | 0.292 | 1 | 0.589 | 0.989 | 0.950 | 1.030 |
| **Age** | -0.055 | 0.019 | 7.973 | 1 | 0.005 | 0.947 | 0.911 | 0.983 |
| **Sex** | 0.199 | 0.297 | 0.451 | 1 | 0.502 | 1.221 | 0.682 | 2.185 |
| **KCL (≥8)** | 1.749 | 0.251 | 48.598 | 1 | 0.000 | 5.749 | 3.516 | 9.400 |

Sex indicates a man against a woman.

**[Below are the results of the analysis for Model 2]**

| **Hosmer and Lemeshow Tests** | | |
| --- | --- | --- |
| χ square | degree of freedom | p-value |
| 5.614 | 8 | 0.690 |

| **Variables in equations** | | | | | | | | |
| --- | --- | --- | --- | --- | --- | --- | --- | --- |
|  | B | SE | Wald | degree of freedom | p-value | Exp(B) | 95% confidence interval | |
|  |  |  |  |  |  |  | minimum | maximum |
| **LSNS-6 score** | -0.108 | 0.020 | 29.630 | 1 | 0.000 | 0.898 | 0.864 | 0.933 |
| **Years of education** | -0.072 | 0.029 | 6.100 | 1 | 0.014 | 0.930 | 0.878 | 0.985 |
| **Number of medications** | 0.037 | 0.032 | 1.310 | 1 | 0.252 | 1.038 | 0.974 | 1.105 |
| **Total physical activity** | -0.013 | 0.007 | 3.739 | 1 | 0.053 | 0.987 | 0.973 | 1.000 |
| **Grip strength** | -0.018 | 0.023 | 0.627 | 1 | 0.428 | 0.982 | 0.938 | 1.027 |
| **Age** | -0.030 | 0.018 | 2.550 | 1 | 0.110 | 0.971 | 0.936 | 1.007 |
| **Sex** | 0.262 | 0.307 | 0.731 | 1 | 0.393 | 1.300 | 0.713 | 2.370 |
| **mCHS (≥3)** | 0.889 | 0.260 | 11.644 | 1 | 0.001 | 2.432 | 1.460 | 4.051 |

Sex indicates a man against a woman.

**[Below are the results of the analysis for Model 3]**

| **Hosmer and Lemeshow Tests** | | |
| --- | --- | --- |
| χ square | degree of freedom | p-value |
| 6.770 | 8 | 0.562 |

| **Variables in equations** | | | | | | | | |
| --- | --- | --- | --- | --- | --- | --- | --- | --- |
|  | B | SE | Wald | degree of freedom | p-value | Exp(B) | 95% confidence interval | |
|  |  |  |  |  |  |  | minimum | maximum |
| **LSNS-6 score** | -0.107 | 0.019 | 30.218 | 1 | 0.000 | 0.899 | 0.865 | 0.934 |
| **Years of education** | -0.078 | 0.028 | 7.540 | 1 | 0.006 | 0.925 | 0.875 | 0.978 |
| **Number of medications** | 0.044 | 0.032 | 1.905 | 1 | 0.167 | 1.045 | 0.982 | 1.111 |
| **Total physical activity** | -0.016 | 0.007 | 5.203 | 1 | 0.023 | 0.984 | 0.971 | 0.998 |
| **Grip strength** | -0.020 | 0.020 | 1.022 | 1 | 0.312 | 0.980 | 0.944 | 1.019 |
| **Age** | -0.027 | 0.018 | 2.227 | 1 | 0.136 | 0.973 | 0.939 | 1.009 |
| **Sex** | 0.296 | 0.281 | 1.104 | 1 | 0.293 | 1.344 | 0.774 | 2.332 |
| **CFS (≥4)** | 0.459 | 0.229 | 4.012 | 1 | 0.045 | 1.582 | 1.010 | 2.478 |

Sex indicates a man against a woman.

**Results of detailed binomial logistic regression analysis in Table 3**

**Models 1, 2, and 3 with depressive symptoms as the dependent variable in patients with diabetes mellitus are as follows.**

**[Below are the results of the analysis for Model 1]**

| **Hosmer and Lemeshow Tests** | | |
| --- | --- | --- |
| χ square | degree of freedom | p-value |
| 7.480 | 8 | 0.486 |

| **Variables in equations** | | | | | | | | |
| --- | --- | --- | --- | --- | --- | --- | --- | --- |
|  | B | SE | Wald | degree of freedom | p-value | Exp(B) | 95% confidence interval | |
|  |  |  |  |  |  |  | minimum | maximum |
| **LSNS-6 score** | -0.069 | 0.025 | 7.725 | 1 | 0.005 | 0.934 | 0.890 | 0.980 |
| **Years of education** | -0.055 | 0.037 | 2.228 | 1 | 0.136 | 0.946 | 0.880 | 1.018 |
| **Number of medications** | 0.021 | 0.041 | 0.256 | 1 | 0.613 | 1.021 | 0.942 | 1.107 |
| **Total physical activity** | -0.012 | 0.008 | 2.467 | 1 | 0.116 | 0.988 | 0.972 | 1.003 |
| **Grip strength** | -0.033 | 0.026 | 1.638 | 1 | 0.201 | 0.968 | 0.921 | 1.018 |
| **Age** | -0.062 | 0.025 | 5.991 | 1 | 0.014 | 0.940 | 0.894 | 0.988 |
| **Sex** | -0.035 | 0.369 | 0.009 | 1 | 0.923 | 0.965 | 0.469 | 1.988 |
| **KCL (≥8)** | 1.946 | 0.309 | 39.699 | 1 | 0.000 | 6.997 | 3.820 | 12.816 |

Sex indicates a man against a woman.

**[Below are the results of the analysis for Model 2]**

| **Hosmer and Lemeshow Tests** | | |
| --- | --- | --- |
| χ square | degree of freedom | p-value |
| 6.645 | 8 | 0.575 |

| **Variables in equations** | | | | | | | | |
| --- | --- | --- | --- | --- | --- | --- | --- | --- |
|  | B | SE | Wald | degree of freedom | p-value | Exp(B) | 95% confidence interval | |
|  |  |  |  |  |  |  | minimum | maximum |
| **LSNS-6 score** | -0.085 | 0.023 | 13.080 | 1 | 0.000 | 0.919 | 0.878 | 0.962 |
| **Years of education** | -0.083 | 0.036 | 5.366 | 1 | 0.021 | 0.920 | 0.858 | 0.987 |
| **Number of medications** | 0.042 | 0.040 | 1.114 | 1 | 0.291 | 1.043 | 0.965 | 1.127 |
| **Total physical activity** | -0.015 | 0.008 | 3.470 | 1 | 0.063 | 0.986 | 0.971 | 1.001 |
| **Grip strength** | -0.053 | 0.026 | 4.116 | 1 | 0.042 | 0.948 | 0.901 | 0.998 |
| **Age** | -0.036 | 0.023 | 2.368 | 1 | 0.124 | 0.965 | 0.922 | 1.010 |
| **Sex** | 0.209 | 0.354 | 0.348 | 1 | 0.555 | 1.232 | 0.616 | 2.465 |
| **mCHS (≥3)** | 0.559 | 0.318 | 3.098 | 1 | 0.078 | 1.749 | 0.939 | 3.260 |

Sex indicates a man against a woman.

**[Below are the results of the analysis for Model 3]**

| **Hosmer and Lemeshow Tests** | | |
| --- | --- | --- |
| χ square | degree of freedom | p-value |
| 7.792 | 8 | 0.454 |

| **Variables in equations** | | | | | | | | |
| --- | --- | --- | --- | --- | --- | --- | --- | --- |
|  | B | SE | Wald | degree of freedom | p-value | Exp(B) | 95% confidence interval | |
|  |  |  |  |  |  |  | minimum | maximum |
| **LSNS-6 score** | -0.083 | 0.023 | 13.106 | 1 | 0.000 | 0.920 | 0.880 | 0.963 |
| **Years of education** | -0.081 | 0.035 | 5.405 | 1 | 0.020 | 0.923 | 0.862 | 0.987 |
| **Number of medications** | 0.041 | 0.038 | 1.159 | 1 | 0.282 | 1.042 | 0.967 | 1.123 |
| **Total physical activity** | -0.017 | 0.008 | 4.523 | 1 | 0.033 | 0.983 | 0.968 | 0.999 |
| **Grip strength** | -0.041 | 0.024 | 2.959 | 1 | 0.085 | 0.960 | 0.916 | 1.006 |
| **Age** | -0.033 | 0.023 | 2.128 | 1 | 0.145 | 0.967 | 0.925 | 1.012 |
| **Sex** | 0.067 | 0.339 | 0.040 | 1 | 0.842 | 1.070 | 0.550 | 2.080 |
| **CFS (≥4)** | 0.300 | 0.271 | 1.223 | 1 | 0.269 | 1.350 | 0.793 | 2.299 |

Sex indicates a man against a woman.

**Results of detailed binomial logistic regression analysis in Table 3**

**Models 1, 2, and 3 with depressive symptoms as the dependent variable in patients with atrial fibrillation are as follows.**

**[Below are the results of the analysis for Model 1]**

| **Hosmer and Lemeshow Tests** | | |
| --- | --- | --- |
| χ square | degree of freedom | p-value |
| 5.511 | 8 | 0.702 |

| **Variables in equations** | | | | | | | | |
| --- | --- | --- | --- | --- | --- | --- | --- | --- |
|  | B | SE | Wald | degree of freedom | p-value | Exp(B) | 95% confidence interval | |
|  |  |  |  |  |  |  | minimum | maximum |
| **LSNS-6 score** | -0.112 | 0.064 | 3.098 | 1 | 0.078 | 0.894 | 0.789 | 1.013 |
| **Years of education** | -0.031 | 0.083 | 0.141 | 1 | 0.708 | 0.969 | 0.824 | 1.141 |
| **Number of medications** | 0.070 | 0.105 | 0.446 | 1 | 0.504 | 1.073 | 0.873 | 1.317 |
| **Total physical activity** | -0.013 | 0.036 | 0.124 | 1 | 0.725 | 0.987 | 0.919 | 1.060 |
| **Grip strength** | 0.012 | 0.060 | 0.040 | 1 | 0.842 | 1.012 | 0.900 | 1.139 |
| **Age** | 0.013 | 0.053 | 0.064 | 1 | 0.800 | 1.014 | 0.913 | 1.125 |
| **Sex** | -0.086 | 0.785 | 0.012 | 1 | 0.913 | 0.918 | 0.197 | 4.270 |
| **KCL (≥8)** | 1.636 | 0.680 | 5.782 | 1 | 0.016 | 5.135 | 1.353 | 19.482 |

Sex indicates a man against a woman.

**[Below are the results of the analysis for Model 2]**

| **Hosmer and Lemeshow Tests** | | |
| --- | --- | --- |
| χ square | degree of freedom | p-value |
| 7.877 | 7 | 0.344 |

| **Variables in equations** | | | | | | | | |
| --- | --- | --- | --- | --- | --- | --- | --- | --- |
|  | B | SE | Wald | degree of freedom | p-value | Exp(B) | 95% confidence interval | |
|  |  |  |  |  |  |  | minimum | maximum |
| **LSNS-6 score** | -0.150 | 0.062 | 5.952 | 1 | 0.015 | 0.860 | 0.762 | 0.971 |
| **Years of education** | -0.019 | 0.085 | 0.051 | 1 | 0.822 | 0.981 | 0.831 | 1.158 |
| **Number of medications** | 0.110 | 0.100 | 1.205 | 1 | 0.272 | 1.116 | 0.917 | 1.359 |
| **Total physical activity** | -0.008 | 0.034 | 0.054 | 1 | 0.816 | 0.992 | 0.927 | 1.061 |
| **Grip strength** | 0.086 | 0.075 | 1.312 | 1 | 0.252 | 1.090 | 0.941 | 1.262 |
| **Age** | 0.039 | 0.056 | 0.504 | 1 | 0.478 | 1.040 | 0.933 | 1.160 |
| **Sex** | -0.884 | 0.886 | 0.996 | 1 | 0.318 | 0.413 | 0.073 | 2.346 |
| **mCHS (≥3)** | 1.692 | 0.759 | 4.966 | 1 | 0.026 | 5.430 | 1.226 | 24.052 |

Sex indicates a man against a woman.

**[Below are the results of the analysis for Model 3]**

| **Hosmer and Lemeshow Tests** | | |
| --- | --- | --- |
| χ square | degree of freedom | p-value |
| 6.835 | 8 | 0.554 |

| **Variables in equations** | | | | | | | | |
| --- | --- | --- | --- | --- | --- | --- | --- | --- |
|  | B | SE | Wald | degree of freedom | p-value | Exp(B) | 95% confidence interval | |
|  |  |  |  |  |  |  | minimum | maximum |
| **LSNS-6 score** | -0.159 | 0.058 | 7.527 | 1 | 0.006 | 0.853 | 0.762 | 0.956 |
| **Years of education** | -0.014 | 0.081 | 0.032 | 1 | 0.859 | 0.986 | 0.841 | 1.155 |
| **Number of medications** | 0.124 | 0.105 | 1.397 | 1 | 0.237 | 1.132 | 0.922 | 1.391 |
| **Total physical activity** | -0.021 | 0.034 | 0.378 | 1 | 0.539 | 0.979 | 0.915 | 1.047 |
| **Grip strength** | 0.020 | 0.055 | 0.130 | 1 | 0.718 | 1.020 | 0.915 | 1.137 |
| **Age** | 0.013 | 0.052 | 0.068 | 1 | 0.794 | 1.014 | 0.916 | 1.122 |
| **Sex** | -0.337 | 0.746 | 0.204 | 1 | 0.652 | 0.714 | 0.165 | 3.084 |
| **CFS (≥4)** | 0.403 | 0.634 | 0.405 | 1 | 0.525 | 1.496 | 0.432 | 5.181 |

Sex indicates a man against a woman.

**Results of detailed binomial logistic regression analysis in Table 4**

**Below is a logistic regression analysis in total patients with KCL, mCHS and CFS as the dependent variable.**

**[Analysis results with KCL as the dependent variable]**

| **Hosmer and Lemeshow Tests** | | |
| --- | --- | --- |
| χ square | degree of freedom | p-value |
| 3.861 | 8 | 0.869 |

| **Variables in equations** | | | | | | | | |
| --- | --- | --- | --- | --- | --- | --- | --- | --- |
|  | B | SE | Wald | degree of freedom | p-value | Exp(B) | 95% confidence interval | |
|  |  |  |  |  |  |  | minimum | maximum |
| **Depressive symptoms** | 1.837 | 0.214 | 73.609 | 1 | 0.000 | 6.278 | 4.126 | 9.551 |
| **Age** | 0.105 | 0.017 | 36.439 | 1 | 0.000 | 1.111 | 1.074 | 1.150 |
| **Sex** | -0.204 | 0.222 | 0.847 | 1 | 0.357 | 0.815 | 0.528 | 1.259 |
| **LSNS-6 score** | -0.069 | 0.019 | 12.937 | 1 | 0.000 | 0.933 | 0.899 | 0.969 |
| **Years of education** | -0.021 | 0.029 | 0.529 | 1 | 0.467 | 0.979 | 0.926 | 1.036 |
| **Number of medication** | 0.064 | 0.034 | 3.406 | 1 | 0.065 | 1.066 | 0.996 | 1.140 |
| **Total number of cadiometabolic diseases** | -0.023 | 0.111 | 0.044 | 1 | 0.833 | 0.977 | 0.787 | 1.213 |
| **Total physical activity** | -0.022 | 0.008 | 7.734 | 1 | 0.005 | 0.978 | 0.963 | 0.993 |
| **BMI** | -0.002 | 0.027 | 0.008 | 1 | 0.930 | 0.998 | 0.946 | 1.052 |

Sex indicates a man against a woman.

**[Analysis results with mCHS as the dependent variable]**

| **Hosmer and Lemeshow Tests** | | |
| --- | --- | --- |
| χ square | degree of freedom | p-value |
| 6.537 | 8 | 0.587 |

| **Variables in equations** | | | | | | | | |
| --- | --- | --- | --- | --- | --- | --- | --- | --- |
|  | B | SE | Wald | degree of freedom | p-value | Exp(B) | 95% confidence interval | |
|  |  |  |  |  |  |  | minimum | maximum |
| **Depressive symptoms** | 0.979 | 0.229 | 18.282 | 1 | 0.000 | 2.661 | 1.699 | 4.167 |
| **Age** | 0.090 | 0.018 | 24.313 | 1 | 0.000 | 1.094 | 1.056 | 1.134 |
| **Sex** | -0.456 | 0.240 | 3.624 | 1 | 0.057 | 0.634 | 0.396 | 1.014 |
| **LSNS-6 score** | -0.038 | 0.020 | 3.507 | 1 | 0.061 | 0.963 | 0.925 | 1.002 |
| **Years of education** | 0.001 | 0.030 | 0.001 | 1 | 0.973 | 1.001 | 0.943 | 1.062 |
| **Number of medication** | 0.096 | 0.036 | 7.288 | 1 | 0.007 | 1.101 | 1.027 | 1.181 |
| **Total number of cadiometabolic diseases** | 0.009 | 0.114 | 0.006 | 1 | 0.937 | 1.009 | 0.807 | 1.262 |
| **Total physical activity** | -0.036 | 0.011 | 11.376 | 1 | 0.001 | 0.964 | 0.944 | 0.985 |
| **BMI** | -0.009 | 0.028 | 0.090 | 1 | 0.764 | 0.992 | 0.938 | 1.048 |

Sex indicates a man against a woman.

**[Analysis results with CFS as the dependent variable]**

| **Hosmer and Lemeshow Tests** | | |
| --- | --- | --- |
| χ square | degree of freedom | p-value |
| 14.003 | 8 | 0.082 |

| **Variables in equations** | | | | | | | | |
| --- | --- | --- | --- | --- | --- | --- | --- | --- |
|  | B | SE | Wald | degree of freedom | p-value | Exp(B) | 95% confidence interval | |
|  |  |  |  |  |  |  | minimum | maximum |
| **Depressive symptoms** | 0.263 | 0.200 | 1.731 | 1 | 0.188 | 1.301 | 0.879 | 1.925 |
| **Age** | 0.092 | 0.016 | 32.671 | 1 | 0.000 | 1.096 | 1.062 | 1.131 |
| **Sex** | -0.418 | 0.205 | 4.137 | 1 | 0.042 | 0.659 | 0.440 | 0.985 |
| **LSNS-6 score** | -0.050 | 0.018 | 7.918 | 1 | 0.005 | 0.952 | 0.919 | 0.985 |
| **Years of education** | 0.037 | 0.027 | 1.838 | 1 | 0.175 | 1.038 | 0.984 | 1.094 |
| **Number of medication** | 0.080 | 0.032 | 6.439 | 1 | 0.011 | 1.084 | 1.018 | 1.153 |
| **Total number of cadiometabolic diseases** | 0.026 | 0.101 | 0.066 | 1 | 0.797 | 1.026 | 0.842 | 1.252 |
| **Total physical activity** | -0.039 | 0.009 | 19.447 | 1 | 0.000 | 0.961 | 0.945 | 0.978 |
| **BMI** | 0.033 | 0.025 | 1.673 | 1 | 0.196 | 1.034 | 0.983 | 1.086 |

Sex indicates a man against a woman.

**Results of detailed binomial logistic regression analysis in Table 4**

**Below is a logistic regression analysis in hypertensive patients with KCL, mCHS and CFS as the dependent variable.**

**[Analysis results with KCL as the dependent variable]**

| **Hosmer and Lemeshow Tests** | | |
| --- | --- | --- |
| χ square | degree of freedom | p-value |
| 6.651 | 8 | 0.575 |

| **Variables in equations** | | | | | | | | |
| --- | --- | --- | --- | --- | --- | --- | --- | --- |
|  | B | SE | Wald | degree of freedom | p-value | Exp(B) | 95% confidence interval | |
|  |  |  |  |  |  |  | minimum | maximum |
| **Depressive symptoms** | 1.719 | 0.255 | 45.537 | 1 | 0.000 | 5.577 | 3.385 | 9.187 |
| **Age** | 0.114 | 0.021 | 29.309 | 1 | 0.000 | 1.121 | 1.076 | 1.168 |
| **Sex** | -0.139 | 0.262 | 0.281 | 1 | 0.596 | 0.870 | 0.521 | 1.455 |
| **LSNS-6 score** | -0.087 | 0.023 | 13.952 | 1 | 0.000 | 0.917 | 0.876 | 0.959 |
| **Years of education** | -0.070 | 0.034 | 4.361 | 1 | 0.037 | 0.932 | 0.872 | 0.996 |
| **Number of medication** | 0.049 | 0.040 | 1.478 | 1 | 0.224 | 1.050 | 0.970 | 1.137 |
| **Total number of cadiometabolic diseases** | -0.020 | 0.143 | 0.019 | 1 | 0.889 | 0.980 | 0.741 | 1.296 |
| **Total physical activity** | -0.028 | 0.010 | 8.611 | 1 | 0.003 | 0.972 | 0.954 | 0.991 |
| **BMI** | -0.002 | 0.031 | 0.005 | 1 | 0.943 | 0.998 | 0.939 | 1.060 |

Sex indicates a man against a woman.

**[Analysis results with mCHS as the dependent variable]**

| **Hosmer and Lemeshow Tests** | | |
| --- | --- | --- |
| χ square | degree of freedom | p-value |
| 3.330 | 8 | 0.912 |

| **Variables in equations** | | | | | | | | |
| --- | --- | --- | --- | --- | --- | --- | --- | --- |
|  | B | SE | Wald | degree of freedom | p-value | Exp(B) | 95% confidence interval | |
|  |  |  |  |  |  |  | minimum | maximum |
| **Depressive symptoms** | 0.868 | 0.262 | 10.960 | 1 | 0.001 | 2.383 | 1.425 | 3.985 |
| **Age** | 0.096 | 0.021 | 21.135 | 1 | 0.000 | 1.100 | 1.056 | 1.146 |
| **Sex** | -0.211 | 0.266 | 0.628 | 1 | 0.428 | 0.810 | 0.480 | 1.364 |
| **LSNS-6 score** | -0.019 | 0.023 | 0.677 | 1 | 0.411 | 0.981 | 0.938 | 1.027 |
| **Years of education** | -0.018 | 0.034 | 0.292 | 1 | 0.589 | 0.982 | 0.918 | 1.049 |
| **Number of medication** | 0.088 | 0.040 | 4.810 | 1 | 0.028 | 1.092 | 1.009 | 1.182 |
| **Total number of cadiometabolic diseases** | -0.118 | 0.142 | 0.693 | 1 | 0.405 | 0.889 | 0.673 | 1.173 |
| **Total physical activity** | -0.039 | 0.012 | 10.463 | 1 | 0.001 | 0.961 | 0.939 | 0.985 |
| **BMI** | -0.026 | 0.032 | 0.663 | 1 | 0.416 | 0.975 | 0.916 | 1.037 |

Sex indicates a man against a woman.

**[Analysis results with CFS as the dependent variable]**

| **Hosmer and Lemeshow Tests** | | |
| --- | --- | --- |
| χ square | degree of freedom | p-value |
| 3.639 | 8 | 0.888 |

| **Variables in equations** | | | | | | | | |
| --- | --- | --- | --- | --- | --- | --- | --- | --- |
|  | B | SE | Wald | degree of freedom | p-value | Exp(B) | 95% confidence interval | |
|  |  |  |  |  |  |  | minimum | maximum |
| **Depressive symptoms** | 0.448 | 0.235 | 3.624 | 1 | 0.057 | 1.565 | 0.987 | 2.483 |
| **Age** | 0.101 | 0.019 | 27.814 | 1 | 0.000 | 1.106 | 1.066 | 1.149 |
| **Sex** | -0.494 | 0.239 | 4.262 | 1 | 0.039 | 0.610 | 0.382 | 0.975 |
| **LSNS-6 score** | -0.038 | 0.021 | 3.314 | 1 | 0.069 | 0.963 | 0.925 | 1.003 |
| **Years of education** | 0.033 | 0.031 | 1.135 | 1 | 0.287 | 1.034 | 0.973 | 1.099 |
| **Number of medication** | 0.083 | 0.037 | 5.067 | 1 | 0.024 | 1.086 | 1.011 | 1.167 |
| **Total number of cadiometabolic diseases** | 0.056 | 0.129 | 0.189 | 1 | 0.664 | 1.057 | 0.822 | 1.361 |
| **Total physical activity** | -0.037 | 0.010 | 13.744 | 1 | 0.000 | 0.964 | 0.945 | 0.983 |
| **BMI** | 0.029 | 0.029 | 1.027 | 1 | 0.311 | 1.030 | 0.973 | 1.090 |

Sex indicates a man against a woman.

**Results of detailed binomial logistic regression analysis in Table 4**

**Below is a logistic regression analysis in patients with diabetes mellitus with KCL, mCHS and CFS as the dependent variable.**

**[Analysis results with KCL as the dependent variable]**

| **Hosmer and Lemeshow Tests** | | |
| --- | --- | --- |
| χ square | degree of freedom | p-value |
| 8.482 | 8 | 0.388 |

| **Variables in equations** | | | | | | | | |
| --- | --- | --- | --- | --- | --- | --- | --- | --- |
|  | B | SE | Wald | degree of freedom | p-value | Exp(B) | 95% confidence interval | |
|  |  |  |  |  |  |  | minimum | maximum |
| **Depressive symptoms** | 2.000 | 0.314 | 40.549 | 1 | 0.000 | 7.388 | 3.992 | 13.674 |
| **Age** | 0.103 | 0.026 | 16.148 | 1 | 0.000 | 1.109 | 1.054 | 1.166 |
| **Sex** | 0.013 | 0.310 | 0.002 | 1 | 0.967 | 1.013 | 0.552 | 1.860 |
| **LSNS-6 score** | -0.052 | 0.027 | 3.643 | 1 | 0.056 | 0.949 | 0.899 | 1.001 |
| **Years of education** | -0.067 | 0.043 | 2.447 | 1 | 0.118 | 0.935 | 0.860 | 1.017 |
| **Number of medication** | 0.048 | 0.051 | 0.920 | 1 | 0.338 | 1.050 | 0.951 | 1.159 |
| **Total number of cadiometabolic diseases** | 0.126 | 0.176 | 0.514 | 1 | 0.474 | 1.134 | 0.804 | 1.601 |
| **Total physical activity** | -0.013 | 0.009 | 1.961 | 1 | 0.161 | 0.987 | 0.970 | 1.005 |
| **BMI** | 0.042 | 0.044 | 0.933 | 1 | 0.334 | 1.043 | 0.958 | 1.136 |

Sex indicates a man against a woman.

**[Analysis results with mCHS as the dependent variable]**

| **Hosmer and Lemeshow Tests** | | |
| --- | --- | --- |
| χ square | degree of freedom | p-value |
| 11.355 | 8 | 0.182 |

| **Variables in equations** | | | | | | | | |
| --- | --- | --- | --- | --- | --- | --- | --- | --- |
|  | B | SE | Wald | degree of freedom | p-value | Exp(B) | 95% confidence interval | |
|  |  |  |  |  |  |  | minimum | maximum |
| **Depressive symptoms** | 0.653 | 0.331 | 3.901 | 1 | 0.048 | 1.921 | 1.005 | 3.672 |
| **Age** | 0.069 | 0.027 | 6.778 | 1 | 0.009 | 1.072 | 1.017 | 1.129 |
| **Sex** | -0.806 | 0.346 | 5.433 | 1 | 0.020 | 0.447 | 0.227 | 0.880 |
| **LSNS-6 score** | -0.051 | 0.029 | 2.988 | 1 | 0.084 | 0.950 | 0.897 | 1.007 |
| **Years of education** | 0.024 | 0.047 | 0.262 | 1 | 0.608 | 1.025 | 0.934 | 1.124 |
| **Number of medication** | 0.078 | 0.052 | 2.247 | 1 | 0.134 | 1.081 | 0.976 | 1.197 |
| **Total number of cadiometabolic diseases** | 0.078 | 0.186 | 0.175 | 1 | 0.675 | 1.081 | 0.751 | 1.555 |
| **Total physical activity** | -0.043 | 0.016 | 7.275 | 1 | 0.007 | 0.958 | 0.928 | 0.988 |
| **BMI** | -0.025 | 0.045 | 0.310 | 1 | 0.578 | 0.975 | 0.892 | 1.066 |

Sex indicates a man against a woman.

**[Analysis results with CFS as the dependent variable]**

| **Hosmer and Lemeshow Tests** | | |
| --- | --- | --- |
| χ square | degree of freedom | p-value |
| 30.909 | 8 | 0.000 |

| **Variables in equations** | | | | | | | | |
| --- | --- | --- | --- | --- | --- | --- | --- | --- |
|  | B | SE | Wald | degree of freedom | p-value | Exp(B) | 95% confidence interval | |
|  |  |  |  |  |  |  | minimum | maximum |
| **Depressive symptoms** | 0.275 | 0.283 | 0.949 | 1 | 0.330 | 1.317 | 0.757 | 2.293 |
| **Age** | 0.076 | 0.023 | 10.945 | 1 | 0.001 | 1.079 | 1.032 | 1.129 |
| **Sex** | -0.826 | 0.292 | 8.017 | 1 | 0.005 | 0.438 | 0.247 | 0.775 |
| **LSNS-6 score** | -0.048 | 0.025 | 3.814 | 1 | 0.051 | 0.953 | 0.908 | 1.000 |
| **Years of education** | 0.052 | 0.039 | 1.728 | 1 | 0.189 | 1.053 | 0.975 | 1.137 |
| **Number of medication** | 0.025 | 0.044 | 0.330 | 1 | 0.565 | 1.026 | 0.941 | 1.118 |
| **Total number of cadiometabolic diseases** | 0.212 | 0.158 | 1.782 | 1 | 0.182 | 1.236 | 0.906 | 1.686 |
| **Total physical activity** | -0.035 | 0.011 | 9.662 | 1 | 0.002 | 0.966 | 0.945 | 0.987 |
| **BMI** | 0.025 | 0.040 | 0.416 | 1 | 0.519 | 1.026 | 0.949 | 1.108 |

Sex indicates a man against a woman.

**Results of detailed binomial logistic regression analysis in Table 4**

**Below is a logistic regression analysis in patients with atrial fibrillation with KCL, mCHS and CFS as the dependent variable**

**[Analysis results with KCL as the dependent variable]**

| **Hosmer and Lemeshow Tests** | | |
| --- | --- | --- |
| χ square | degree of freedom | p-value |
| 6.194 | 7 | 0.517 |

| **Variables in equations** | | | | | | | | |
| --- | --- | --- | --- | --- | --- | --- | --- | --- |
|  | B | SE | Wald | degree of freedom | p-value | Exp(B) | 95% confidence interval | |
|  |  |  |  |  |  |  | minimum | maximum |
| **Depressive symptoms** | 1.566 | 0.718 | 4.755 | 1 | 0.029 | 4.786 | 1.172 | 19.552 |
| **Age** | 0.009 | 0.059 | 0.024 | 1 | 0.876 | 1.009 | 0.899 | 1.133 |
| **Sex** | -0.759 | 0.806 | 0.888 | 1 | 0.346 | 0.468 | 0.096 | 2.270 |
| **LSNS-6 score** | -0.195 | 0.071 | 7.448 | 1 | 0.006 | 0.823 | 0.716 | 0.947 |
| **Years of education** | 0.092 | 0.099 | 0.862 | 1 | 0.353 | 1.096 | 0.903 | 1.332 |
| **Number of medication** | 0.229 | 0.140 | 2.689 | 1 | 0.101 | 1.257 | 0.956 | 1.653 |
| **Total number of cadiometabolic diseases** | 0.357 | 0.368 | 0.940 | 1 | 0.332 | 1.429 | 0.695 | 2.938 |
| **Total physical activity** | -0.039 | 0.040 | 0.964 | 1 | 0.326 | 0.962 | 0.889 | 1.040 |
| **BMI** | 0.035 | 0.102 | 0.116 | 1 | 0.734 | 1.035 | 0.848 | 1.264 |

Sex indicates a man against a woman.

**[Analysis results with mCHS as the dependent variable]**

| **Hosmer and Lemeshow Tests** | | |
| --- | --- | --- |
| χ square | degree of freedom | p-value |
| 8.721 | 8 | 0.366 |

| **Variables in equations** | | | | | | | | |
| --- | --- | --- | --- | --- | --- | --- | --- | --- |
|  | B | SE | Wald | degree of freedom | p-value | Exp(B) | 95% confidence interval | |
|  |  |  |  |  |  |  | minimum | maximum |
| **Depressive symptoms** | 1.354 | 0.672 | 4.058 | 1 | 0.044 | 3.874 | 1.037 | 14.472 |
| **Age** | -0.024 | 0.058 | 0.173 | 1 | 0.677 | 0.976 | 0.871 | 1.094 |
| **Sex** | -0.266 | 0.754 | 0.124 | 1 | 0.725 | 0.767 | 0.175 | 3.364 |
| **LSNS-6 score** | -0.055 | 0.064 | 0.731 | 1 | 0.393 | 0.947 | 0.835 | 1.073 |
| **Years of education** | 0.001 | 0.088 | 0.000 | 1 | 0.992 | 1.001 | 0.842 | 1.190 |
| **Number of medication** | 0.184 | 0.128 | 2.061 | 1 | 0.151 | 1.202 | 0.935 | 1.544 |
| **Total number of cadiometabolic diseases** | 0.161 | 0.354 | 0.206 | 1 | 0.650 | 1.174 | 0.586 | 2.352 |
| **Total physical activity** | -0.067 | 0.045 | 2.180 | 1 | 0.140 | 0.936 | 0.856 | 1.022 |
| **BMI** | -0.051 | 0.099 | 0.267 | 1 | 0.605 | 0.950 | 0.783 | 1.153 |

Sex indicates a man against a woman.

**[Analysis results with CFS as the dependent variable]**

| **Hosmer and Lemeshow Tests** | | |
| --- | --- | --- |
| χ square | degree of freedom | p-value |
| 8.961 | 7 | 0.255 |

| **Variables in equations** | | | | | | | | |
| --- | --- | --- | --- | --- | --- | --- | --- | --- |
|  | B | SE | Wald | degree of freedom | p-value | Exp(B) | 95% confidence interval | |
|  |  |  |  |  |  |  | minimum | maximum |
| **Depressive symptoms** | 0.185 | 0.692 | 0.072 | 1 | 0.789 | 1.203 | 0.310 | 4.667 |
| **Age** | 0.072 | 0.053 | 1.857 | 1 | 0.173 | 1.075 | 0.969 | 1.193 |
| **Sex** | -0.091 | 0.722 | 0.016 | 1 | 0.899 | 0.913 | 0.222 | 3.756 |
| **LSNS-6 score** | -0.076 | 0.059 | 1.651 | 1 | 0.199 | 0.926 | 0.825 | 1.041 |
| **Years of education** | 0.108 | 0.085 | 1.635 | 1 | 0.201 | 1.114 | 0.944 | 1.316 |
| **Number of medication** | 0.215 | 0.128 | 2.831 | 1 | 0.092 | 1.240 | 0.965 | 1.593 |
| **Total number of cadiometabolic diseases** | 0.697 | 0.370 | 3.550 | 1 | 0.060 | 2.008 | 0.972 | 4.148 |
| **Total physical activity** | -0.042 | 0.039 | 1.148 | 1 | 0.284 | 0.959 | 0.888 | 1.036 |
| **BMI** | 0.153 | 0.103 | 2.202 | 1 | 0.138 | 1.165 | 0.952 | 1.426 |

Sex indicates a man against a woman.
